# MicroRNA profiling in adults with high-functioning autism spectrum disorder

**DOI:** 10.1186/s13041-019-0508-6

**Published:** 2019-10-21

**Authors:** Masatoshi Nakata, Ryo Kimura, Yasuko Funabiki, Tomonari Awaya, Toshiya Murai, Masatoshi Hagiwara

**Affiliations:** 1 Department of Anatomy and Developmental Biology, Graduate School of Medicine, Kyoto University, Kyoto, 606-8501 Japan; 2 Department of Cognitive and Behavioral Science, Graduate School of Human and Environmental Studies, Kyoto University, Kyoto, 606-8501 Japan; 3 Department of Psychiatry, Graduate School of Medicine, Kyoto University, Kyoto, 606-8507 Japan

**Keywords:** Autism spectrum disorder, High-functioning, Microarray, MicroRNA

## Abstract

Autism spectrum disorder (ASD) is a neurodevelopmental disorder characterized by social communication deficits and repetitive behaviors. Owing to the difficulty of clinical diagnosis, ASD without intellectual disability (i.e., high-functioning ASD) is often overlooked. MicroRNAs (miRNAs) have been recently recognized as potential biomarkers of ASD as they are dysregulated in various tissues of individuals with ASD. However, it remains unclear whether miRNA expression is altered in individuals with high-functioning ASD. Here, we investigated the miRNA expression profile in peripheral blood from adults with high-functioning ASD, and age and gender-matched healthy controls. We identified miR-6126 as being robustly down-regulated in ASD and correlated with the severity of social deficits. Enrichment analysis of predicted target genes revealed potential association with neurons, synapses, and oxytocin signaling pathways. Our findings may provide insights regarding the molecular clues for recognizing high-functioning ASD.

Autism spectrum disorder (ASD) is a neurodevelopmental disorder characterized by social communication deficits and repetitive behaviors [1]. ASD diagnosis is currently based on clinical symptoms. However, the objective and quantitative assessment of sociality can be challenging during the limited time commonly available in clinical appointments. Additionally, it has recently been recognized that a substantial number of cases of ASD are overlooked, even until adulthood [2]. This may occur because the diagnosis of ASD is usually made in early childhood concomitant with evident symptoms; conversely, recognition of cases with less severe symptoms and/or without associated intellectual disability (ID), such as those with average or above-average intelligence quotients (IQ) (full-scale IQ > 70; i.e., high-functioning ASD) is likely to be delayed. Furthermore, these late-diagnosed individuals tend to suffer from mental health problems related to long-term stress from daily life [2, 3], highlighting the needed for accurate diagnosis and adequate support.

Numerous studies have attempted to identify biomarkers to facilitate ASD diagnosis. For example, microRNAs (miRNAs), which comprise small non-coding RNAs that participate in post-transcriptional regulation of gene expression, constitute a potential biomarker in psychiatric disorders owing to their advantage of stability [4]. Accumulating evidence supports that miRNA expression profiles are altered in various tissues of patients with ASD including the brain, blood, and saliva [5]. However, most studies to date have focused on children with classic ASD; to our knowledge, no published studies have reported miRNA alterations in high-functioning ASD [5].

Here, we sought to explore miRNAs in the blood from adults with high-functioning ASD. A flow chart of the study is shown in Fig. 1a. The clinical characteristics of participants are shown in Additional file 1: Table S1. No participants exhibited ID although small albeit significant differences in full and performance IQ scores were noted between subjects with ASD and controls. The Autism Diagnostic Observation Scale and Social Responsiveness Scale (SRS-2) scores that were used to evaluate the severity of ASD were higher in individuals with ASD compared to those in controls, as expected.

**Fig. 1:**
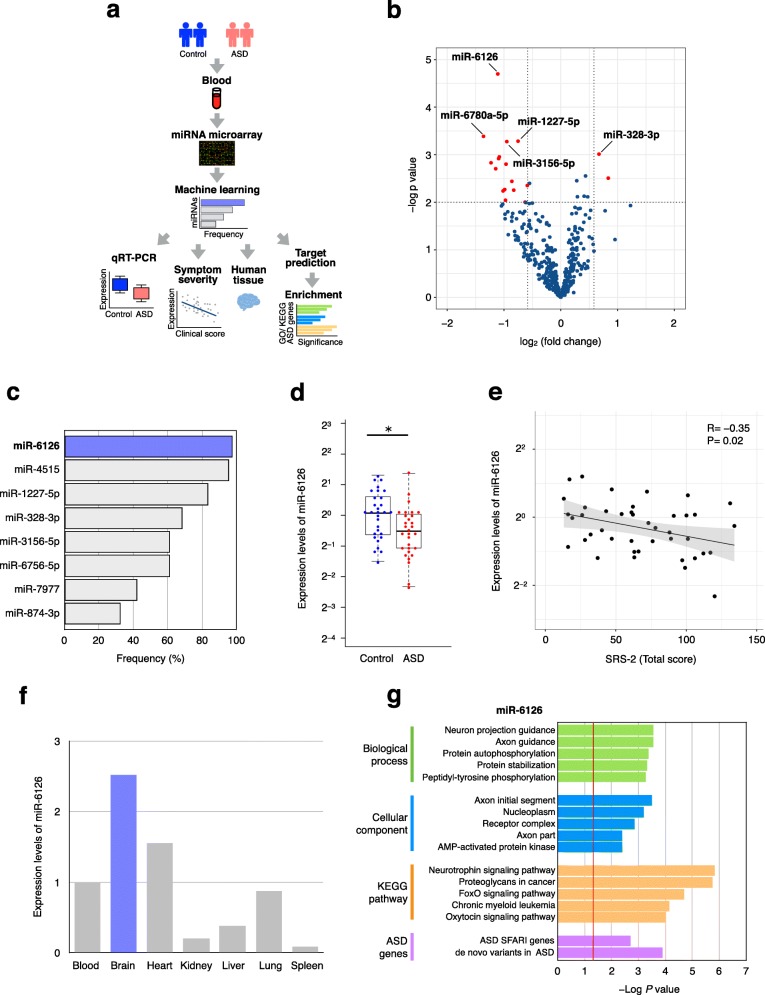
miRNA expression profiles in blood from adults with high-functioning ASD and controls. **a** Flow chart of the study design. **b** Differential miRNA expression profiles determined using microarray. Red dots indicate miRNAs with *p*-values < 0.01 and |fold change| > 1.5. **c** Frequency of miRNA selection was calculated using a greedy algorithm. A total of 2000 selections using random 80% subsets of both ASD and controls were run. The eight most frequently selected miRNAs are shown. **d** Expression level of miR-6126 by qRT–PCR. U6 snRNA was used as an internal control. Data are presented as the upper and lower quantiles and range (box). *p < 0.01 **e** Correlation between miR-6126 expression level and SRS-2 total score. Spearman’s correlation coefficients are shown. **f** Expression level of miR-6126 across human tissues. U6 snRNA was used as an internal control. Relative expression was quantified using the ddCt method. **g** GO/KEGG pathway, and ASD-related gene enrichment analyses of target genes of miR-6126. Red line indicates *p*-values < 0.05

To profile miRNA expression, we performed miRNA microarray analysis as previously described [6]. High-quality RNA with an average RNA integrity number (RIN) of 8.7 was used; quality did not significantly differ between the two groups (Additional file 1: Table S1). We identified 18 miRNAs (2 up-regulated and 16 down-regulated) that exhibited significant change between ASD and control groups (Fig. 1b and Additional file 2: Table S2). In particular, miR-6126, miR-3156-5p, miR-1227-5p, miR-6780a-5p, and miR-328-3p were most significantly dysregulated in ASD. To assess the robustness of our results, we applied differentially expressed miRNAs that were identified by microarray analysis to the Coarse Approximation Linear Function algorithm [7]. Upon analyses of 2000 random selections of subsets representing 80% of individuals, miR-6126 was most frequently selected (Fig. 1c). We subsequently confirmed that miR-6126 was significantly down-regulated (*p* = 7.48E− 3) in ASD using quantitative reverse transcription-polymerase chain reaction (qRT-PCR) (Fig. 1d). Together, these results indicated that miR-6126 may serve as the most promising indicator of ASD status in the blood from subjects with high-functioning adult ASD.

To clarify relationships between the expression of miR-6126 and clinical severity of ASD symptoms, we performed correlation analysis. Notably, the expression of miR-6126 was significantly negative correlated with SRS-2 total score (*r* = − 0.35, *p* = 0.02) (Fig. 1e). In contrast, there was no significant correlation between the expression of miR-6126 and IQ scores (full scale, verbal, and performance) (Additional file 3: Table S3). These results suggested that miR-6126 expression is associated with social severity of ASD, independent from IQ. Next, to examine whether miR-6126 is expressed in brain, we analyzed miR-6126 expression in a variety of normal human tissues. We found that miR-6126 was highly enriched in brain tissue among human organs (Fig. 1f). Furthermore, to investigate the biological significance of miR-6126, we searched predicted target genes using the miRWalk 2.0 database [8] (Additional file 4: Table S4). The obtained 1167 predicted target genes were subjected to Gene Ontology (GO) and Kyoto Encyclopedia of Genes and Genomes (KEGG) pathway enrichment analysis. Notably, our results indicated that target genes of miR-6126 are significantly related to axons, neuron guidance, and oxytocin signaling pathways (Fig. 1g and Additional file 5: Table S5). We also found that miR-6126 targets were significantly enriched for ASD-related genes (Fig. 1g). Notably, target genes included representative ASD-related genes such as *ANK3*, *CACNA2D1*, *NRXN3*, and *PCDH9*. Taken together, these findings provide new insight into the potential association of miR-6126 with social deficits in ASD.

Multiple miRNAs have been proposed as potential biomarkers for children with ASD; however, many results are not consistent across studies [5]. The reasons may be due to different factors such as tissue, RNA quality, and medication status. Accordingly, we carefully controlled potential factors including only high-quality RNA and individuals not receiving medication. To date, miR-6126 has been reported to be associated with non-psychiatric diseases, such as ovarian cancer, diabetic nephropathy, and immune thrombocytopenic purpura [9–11]. Nevertheless, the effects of miR-6126 toward ASD and brain function remain unknown. In addition to the brain, we found that miR-6126 was expressed in the heart to a moderate extent (Fig. 1f). Recent studies have suggested lower heart rate variability in ASD compared to controls [12]. Although further investigations are warranted, our results may provide clues for the underlying mechanisms of potential cardiovascular risks in ASD. Notably, miR-6126 is located on 16p13.3, within a copy number variation region significantly associated with ASD that includes *RBFOX1*, *CREBBP*, *TRAF7,* and *TSC2* genes [13]. Conversely, although several studies have examined miRNA expression in adult ASD postmortem brains [14–16], none have reported altered expression of miR-6126. This may be due to these studies being likely based on older versions of miRBase database that did not include information regarding miR-6126.

Several limitations to our findings should be carefully considered. First, the sample size is relatively small. Second, patients consisted of only Japanese individuals from a single university hospital. Third, several factors might impact miRNA assays. Although we confirmed that the differences in estimated blood cell compositions among groups were not significant (Additional file 6: Figure S1), we could not control for the influence of sample collection time or fasting state. Finally, in this study, the target genes of miR-6126 were predicted based on a database and were subsequently applied for enrichment analysis. To validate these results, an experimental approach will be needed to identify actual targets of miR-6126.

To our knowledge, this is the first pilot study demonstrating the miRNA expression profile of peripheral blood from adults with high-functioning ASD. The identified miR-6126 was down-regulated in ASD, correlated with the severity of social deficits, and had predicted target genes associated with neurons and oxytocin signaling pathways. These findings may provide new insights regarding the molecular clues for recognizing high-functioning ASD. To confirm our findings, further studies with more samples and experimental validation are required.

## Supplementary information

**Additional file 1: Table S1.** Clinical characteristics of participants.

**Additional file 2: Table S2.** Differential miRNA expression profiles.

**Additional file 3: Table S3.** Correlations between miR-6126 expression and IQ scores.

**Additional file 4: Table S4.** Predicted target genes of miR-6126.

**Additional file 5: Table S5.** GO and KEGG pathway analysis of target genes of miR-6126.

**Additional file 6: Figure S1.** Blood cell composition. The proportions of six blood cell subtypes [B cells, CD8 T cells, CD4 T cells, natural killer (NK) cells, monocytes, and granulocytes] were estimated by Houseman’s algorithm using DNA methylation array data. A Student’s t-test was used for statistical analysis.

**Additional file 7.** Materials and Methods.

## Data Availability

The datasets and computer code used in the current study are available from the corresponding author on reasonable request. Methods are presented in Additional file 7.

## Abbreviations

ASD: Autism spectrum disorder
GO: Gene ontology
ID: Intellectual disability
IQ: Intelligence quotient
KEGG: Kyoto encyclopedia of genes and genomes
miRNA: microRNA
qRT-PCR: quantitative reverse transcription-polymerase chain reaction
RIN: RNA integrity number
SRS-2: Social responsiveness scale second edition

## References

[1] Lai MC, Lombardo MV, Baron-Cohen S (2014). Autism. Lancet.

[2] Lai MC, Baron-Cohen S (2015). Identifying the lost generation of adults with autism spectrum conditions. Lancet Psychiatry.

[3] Magiati I, Tay XW, Howlin P (2014). Cognitive, language, social and behavioural outcomes in adults with autism spectrum disorders: a systematic review of longitudinal follow-up studies in adulthood. Clin Psychol Rev.

[4] Issler O, Chen A (2015). Determining the role of microRNAs in psychiatric disorders. Nat Rev Neurosci.

[5] Hicks SD, Middleton FA (2016). A comparative review of microRNA expression patterns in autism spectrum disorder. Front Psychiatry.

[6] Kimura R, Swarup V, Tomiwa K, Gandal MJ, Parikshak NN, Funabiki Y (2019). Integrative network analysis reveals biological pathways associated with Williams syndrome. J Child Psychol Psychiatry.

[7] Jeffries CD, Perkins DO, Chandler SD, Stark T, Yeo E, Addington J (2016). Insights into psychosis risk from leukocyte microRNA expression. Transl Psychiatry.

[8] Dweep H, Gretz N (2015). miRWalk2.0: a comprehensive atlas of microRNA-target interactions. Nat Methods.

[9] Delić D, Eisele C, Schmid R, Baum P, Wiech F, Gerl M (2016). Urinary exosomal miRNA signature in type II diabetic nephropathy patients. PLoS One.

[10] Kanlikilicer P, Rashed MH, Bayraktar R, Mitra R, Ivan C, Aslan B (2016). Ubiquitous release of exosomal tumor suppressor miR-6126 from ovarian cancer cells. Cancer Res.

[11] Zuo B, Zhai J, You L, Zhao Y, Yang J, Weng Z (2017). Plasma microRNAs characterising patients with immune thrombo cytopenic purpura. Thromb Haemost.

[12] Thapa R, Alvares GA, Zaidi TA, Thomas EE, Hickie IB, Park SH (2019). Reduced heart rate variability in adults with autism spectrum disorder. Autism Res.

[13] Sebat J, Lakshmi B, Malhotra D, Troge J, Lese-Martin C, Walsh T (2007). Strong association of de novo copy number mutations with autism. Science.

[14] Ander BP, Barger N, Stamova B, Sharp FR, Schumann CM (2015). Atypical miRNA expression in temporal cortex associated with dysregulation of immune, cell cycle, and other pathways in autism spectrum disorders. Mol Autism.

[15] Mor M, Nardone S, Sams DS, Elliott E (2015). Hypomethylation of miR-142 promoter and upregulation of microRNAs that target the oxytocin receptor gene in the autism prefrontal cortex. Mol Autism.

[16] Wu YE, Parikshak NN, Belgard TG, Geschwind DH (2016). Genome-wide, integrative analysis implicates microRNA dysregulation in autism spectrum disorder. Nat Neurosci.

