# Supplementary material for: MicroRNA profiling in adults with high-functioning autism spectrum disorder
Source: Mol Brain. 2019 Oct 21;12:82. doi: 10.1186/s13041-019-0508-6 (PMC6802322; doi:10.1186/s13041-019-0508-6)
Supplement: Supplementary file 1 — Additional file 1: Table S1. Clinical characteristics of participants. [file 13041_2019_508_MOESM1_ESM.docx]

**Table S1. Clinical characteristics of participants.**

|  | ASD | Control |
| --- | --- | --- |
| Individuals, n | 30 | 30 |
| Age, years, mean | 28.4 (7.3) | 28.4 (8.4) |
| Sex, male (%) | 18 (60.0) | 18 (60.0) |
| RIN | 8.7 (0.1) | 8.7 (0.1) |
| WAIS-Ⅲ |  |  |
| Full scale IQ | 104.1 (17.3) * | 114.9 (12.2) |
| Verbal IQ | 109.5 (2.8) | 116.4 (2.7) |
| Performance IQ | 96.1 (3.7) * | 109.8 (2.2) |
| ADOS |  |  |
| Total score | 7.6 (5.6) * | 1.3 (1.4) |
| SRS-2 |  |  |
| Total score | 88.3 (28.8) * | 41.4 (22.1) |

ASD, Autism Spectrum Disorder; RIN, RNA integrity number; WAIS, Wechsler Adult Intelligence Scale; IQ, Intelligence Quotient; ADOS, Autism Diagnostic Observation Schedule; SRS, Social Responsiveness Scale; The standard deviation is shown in parentheses; **p*<0.05 based on Mann-Whitney U test.
