# Supplementary material for: MicroRNA profiling in adults with high-functioning autism spectrum disorder
Source: Mol Brain. 2019 Oct 21;12:82. doi: 10.1186/s13041-019-0508-6 (PMC6802322; doi:10.1186/s13041-019-0508-6)
Supplement: Supplementary file 2 — Additional file 2: Table S2. Differential miRNA expression profiles. [file 13041_2019_508_MOESM2_ESM.docx]

**Table S2. Differential miRNA expression profiles.**

| miRNA | Agilent probe ID | Log_2_FC | *p-*value | FDR *p*-value |
| --- | --- | --- | --- | --- |
| hsa-miR-6126 | A_25_P00017954 | −1.110 | 2.00E-05 | 7.58E-03 |
| hsa-miR-6780a-5p | A_25_P00018982 | −1.362 | 4.10E-04 | 5.36E-02 |
| hsa-miR-1227-5p | A_25_P00017795 | −0.753 | 5.20E-04 | 5.36E-02 |
| hsa-miR-3156-5p | A_25_P00015875 | −0.951 | 5.30E-04 | 5.36E-02 |
| hsa-miR-328-3p | A_25_P00010262 | 0.673 | 9.70E-04 | 7.00E-02 |
| hsa-miR-4716-3p | A_25_P00017155 | −1.083 | 1.11E-03 | 7.00E-02 |
| hsa-miR-144-3p | A_25_P00012189 | −1.095 | 1.21E-03 | 7.00E-02 |
| hsa-miR-3127-5p | A_25_P00015713 | −1.229 | 1.48E-03 | 7.08E-02 |
| hsa-miR-5581-5p | A_25_P00017676 | −0.965 | 1.58E-03 | 7.08E-02 |
| hsa-miR-6756-5p | A_25_P00018131 | −1.147 | 1.97E-03 | 7.98E-02 |
| hsa-miR-4515 | A_25_P00016993 | 0.836 | 3.11E-03 | 1.05E-01 |
| hsa-miR-6767-5p | A_25_P00018984 | −0.862 | 3.63E-03 | 1.05E-01 |
| hsa-miR-7977 | A_25_P00019078 | −0.590 | 4.44E-03 | 1.12E-01 |
| hsa-miR-4486 | A_25_P00017195 | −0.988 | 5.39E-03 | 1.23E-01 |
| hsa-miR-6734-5p | A_25_P00018979 | −0.829 | 5.56E-03 | 1.23E-01 |
| hsa-miR-4653-3p | A_25_P00017154 | −1.017 | 5.78E-03 | 1.23E-01 |
| hsa-miR-6085 | A_25_P00017977 | −0.975 | 9.07E-03 | 1.59E-01 |
| hsa-miR-874-3p | A_25_P00012918 | −0.629 | 9.93E-03 | 1.59E-01 |

List of miRNAs with *p*-values below 0.01 and |fold change| above 1.5. FC, Fold Change; FDR, false discovery rate.
