# Supplementary material for: MicroRNA profiling in adults with high-functioning autism spectrum disorder
Source: Mol Brain. 2019 Oct 21;12:82. doi: 10.1186/s13041-019-0508-6 (PMC6802322; doi:10.1186/s13041-019-0508-6)
Supplement: Supplementary file 3 — Additional file 3: Table S3. Correlations between miR-6126 expression and IQ scores. [file 13041_2019_508_MOESM3_ESM.docx]

**Table S3. Correlations between miR-6126 expression and IQ scores.**

| WAIS-Ⅲ | Spearman's R | p-value |
| --- | --- | --- |
| Full scale IQ | 0.03 | 0.81 |
| Verbal IQ | 0.09 | 0.50 |
| Performance IQ | 0.06 | 0.66 |

Spearman's correlation coefficients (R) and *p*-value were calculated.
