# Supplementary material for: MicroRNA profiling in adults with high-functioning autism spectrum disorder
Source: Mol Brain. 2019 Oct 21;12:82. doi: 10.1186/s13041-019-0508-6 (PMC6802322; doi:10.1186/s13041-019-0508-6)
Supplement: Supplementary file 4 — Additional file 4: Table S4. Predicted target genes of miR-6126. [file 13041_2019_508_MOESM4_ESM.docx]

**Table S4.** **Predicted target genes of miR-6126.**

| Algorithms | miRWalk, miRanda, RNAhybrid, TargetScan |
| --- | --- |
| Target genes | A1CF, AAK1, AATK, ABCB9, ABCG1, ABCG5, ABI2, ABL1, ACACA, ACAP2, ACAP3, ACTA2, ACTC1, ACTR1A, ACVR2B, ADA, ADAM17, ADAMTS12, ADAMTS4, ADAP2, ADARB1, AFAP1, AFF1, AFF2, AFF3, AGAP1, AGAP2, AGO1, AGPS, AGR3, AHCY, AIG1, AK3, AKNA, AKR1E2, AKT1S1, ALDH1L2, ALDH7A1, ALDOC, ALPK3, AMER1, AMMECR1L, ANGEL1, ANK3, ANKLE2, ANKRD34C, ANKRD52, ANKS6, AP4S1, APC2, APLN, AQP2, ARHGAP27, ARHGEF17, ARHGEF2, ARHGEF39, ARHGEF6, ARID3B, ARID5B, ARIH2, ARL4A, ARPC4-TTLL3, ARRB1, ARRDC2, ARSA, ARX, ASAH2B, ASAP1, ASB1, ASB16, ATF7, ATP1B2, ATP2C1, ATP4A, ATP6V1E1, ATP7A, ATP9B, ATXN1, ATXN1L, ATXN3, ATXN7L3B, AVL9, AWAT2, B4GALNT3, B4GALNT4, B4GALT5, BACE1, BACE2, BACH2, BCL6B, BDH1, BEST1, BFSP1, BHLHB9, BICC1, BID, BIRC3, BLOC1S3, BMPR2, BRAF, BRD4, BRF1, BRI3, BRPF3, BTBD9, BTG1, C10orf105, C10orf99, C12orf23, C15orf32, C17orf103, C17orf51, C19orf44, C1GALT1, C1orf115, C1QTNF3, C22orf24, C22orf46, C2orf44, C6, C7orf65, C8orf46, C9orf139, C9orf170, CA1, CABP2, CACFD1, CACNA1E, CACNA2D1, CACNG4, CACNG8, CALCRL, CAMK2A, CAMK2B, CAMK2D, CAMKK1, CANX, CAP1, CAPN6, CASP10, CASP2, CBFA2T2, CBL, CBR4, CBX2, CBX5, CC2D1B, CCDC122, CCDC134, CCL8, CD180, CD37, CD84, CD99, CDC14B, CDC25A, CDC42SE2, CDCA3, CDKL5, CDT1, CECR2, CENPI, CENPQ, CEP85L, CES3, CFLAR, CHD2, CHMP1A, CHMP1B, CHRND, CHST10, CHST6, CISD2, CLCC1, CLCN5, CLCN6, CLDN11, CLEC16A, CLMN, CLOCK, CMBL, CNDP2, CNEP1R1, COL16A1, COLGALT1, COMTD1, COX10, CPPED1, CPT1A, CREB1, CRHR1, CRTAP, CSF1R, CSMD1, CSNK1G1, CTDSP1, CTNNA3, CTNNBIP1, CTSC, CUL3, CXCL12, CXorf36, CYB561A3, CYP4A11, CYP4B1, CYP8B1, CYTH1, DAAM2, DAB2IP, DAGLA, DAK, DAPK3, DBNDD1, DCAF15, DCAF5, DCC, DCUN1D5, DCX, DDI2, DDX46, DERL3, DGAT1, DGCR14, DGCR8, DHRS11, DIDO1, DISC1, DMRTC1, DMRTC1B, DNAAF3, DOCK5, DPH3, DPP10, DPP9, DRP2, DST, DUSP1, DUSP10, DUSP16, DUSP18, DUSP28, DYNC2LI1, E2F2, EBF4, EDEM3, EEA1, EFCAB14, EFHD1, EFNB3, EGFR, EHD2, EHF, EIF1AD, ELF4, ELMSAN1, ENAH, ENDOV, EPB41, EPB41L1, EPB41L4A, EPHA8, EPM2A, ERC1, ERLIN1, ERN1, ERO1LB, ESYT3, ETNPPL, ETV2, EVI5, EXD3, EXOC3, EXOC6B, F13A1, FABP7, FAM105B, FAM109A, FAM115A, FAM120AOS, FAM129A, FAM134A, FAM160A1, FAM167A, FAM189A1, FAM196A, FAM19A2, FAM73A, FAM73B, FAM78B, FAM86C1, FAM8A1, FANCC, FBLN1, FBXL18, FBXO10, FBXO28, FBXO32, FBXO41, FBXO45, FBXW2, FCRL3, FCRL5, FECH, FGD6, FGFR1, FIBIN, FLT1, FMO1, FMO2, FMO5, FOXJ3, FOXL2, FOXO3, FOXP3, FRAS1, FRS2, FSTL4, FUBP1, FUS, FXR1, FZD10, FZD5, GAB2, GABRA3, GABRG1, GALNT16, GALNT2, GAPT, GAREML, GAS7, GAS8, GBX2, GCA, GCNT2, GDF11, GEN1, GFOD1, GFRA1, GHDC, GID8, GJD3, GLG1, GLTSCR1L, GMDS, GNAI2, GNAL, GNL1, GOLPH3L, GPIHBP1, GPR173, GPR4, GPR63, GPR68, GPR88, GPRIN2, GPX2, GRAMD1B, GRAP2, GRIA1, GRID1, GRIN2A, GRTP1, GUCD1, GUCY1A2, H2AFB1, H2AFB2, H2AFB3, HABP2, HAP1, HAUS4, HDAC9, HDC, HDLBP, HEMK1, HEPHL1, HEPN1, HEXIM1, HGS, HHIPL1, HINT3, HIP1R, HLA-DPB1, HLA-E, HLF, HMBOX1, HNF1A, HNF4A, HPGD, HS2ST1, HTR2A, IBA57, IFFO1, IFNLR1, IGFBP5, IKZF2, IL10RA, IL12B, IL12RB2, IL18BP, IL18R1, IL6ST, ING5, INPP5B, INSR, IPCEF1, IPO8, IQCE, IRAK3, IRAK4, IRF5, IRF6, ISG20L2, ISPD, ISY1-RAB43, ITGB1, ITPRIP, JAG2, JRK, KAAG1, KANSL3, KAT2A, KCMF1, KCNA2, KCNH1, KCNJ2, KCNJ5, KCNQ2, KCNQ3, KCNV1, KCTD16, KCTD18, KDM5A, KDM8, KDSR, KIAA0408, KIAA1045, KIAA1147, KIAA1199, KIAA1244, KIAA1715, KIF17, KIF21B, KIF3B, KIF6, KLF12, KLF13, KLHL25, KLHL26, KLHL3, KLK10, KPNA4, KREMEN1, KRT72, KRT76, KRT83, KSR2, L3MBTL1, LACE1, LAIR1, LAMC1, LAMC3, LAMP2, LARGE, LARP4B, LDB2, LDB3, LDLRAD1, LDLRAD2, LDLRAD4, LDOC1L, LETMD1, LGSN, LHFPL4, LIF, LINC00984, LMO4, LPP, LRCH4, LRP4, LRRC14, LRRC27, LRRC28, LRRC66, LRRTM4, LTN1, LUZP1, LYRM9, MACROD2, MAK16, MAN2A2, MAP2, MAP2K5, MAP2K7, MAP3K11, MAP6D1, MAP9, MAPK11, MAPK13, MAPK14, MAPKBP1, MARCH6, MARK4, MAST4, MAVS, MBD2, MBNL1, MBTPS1, MCC, MCTP2, MDGA1, MDH2, MDM4, MEIS3, METTL10, METTL16, MFSD9, MGAT4A, MICAL2, MICAL3, MIPOL1, MLLT1, MLLT6, MMP11, MMP2, MMP8, MMRN2, MN1, MOCS1, MOCS2, MORN1, MPL, MPP1, MPP2, MPP3, MPPED2, MPRIP, MPZL1, MR1, MRPL49, MRPL52, MRPS25, MRPS26, MRRF, MRVI1, MSL2, MSN, MTF1, MTMR10, MTMR3, MTRR, MXI1, MXRA7, MYO10, MYOZ3, NAA38, NAA50, NAA60, NACC1, NAP1L3, NCBP2, NDE1, NDOR1, NDUFB7, NDUFC2-KCTD14, NEDD4L, NEK6, NEU1, NEURL1B, NFASC, NFATC3, NFX1, NHLRC4, NIPAL3, NIPSNAP1, NKX2-2, NMT1, NMUR1, NOC2L, NOL12, NOL9, NOS3, NOTCH2NL, NPAS2, NR2C2, NR2C2AP, NR2E1, NRAS, NREP, NRL, NRSN1, NRXN3, NSL1, NTRK2, NUB1, NUDT16, NUMA1, NUPL1, NXF1, OGFOD2, OGFOD3, OLR1, ONECUT2, OPCML, OPHN1, OPRL1, OR6B2, ORAOV1, OS9, OSBPL5, OST4, OTUB1, OTUD3, P2RX7, P2RY6, PA2G4, PAGR1, PAPOLG, PATE3, PAX1, PAX6, PBLD, PBX1, PC, PCBP3, PCDH9, PCDHGA11, PCF11, PCM1, PDK3, PDPR, PDRG1, PDXK, PECR, PELI3, PEX16, PEX19, PFKFB2, PGF, PGM3, PGP, PGR, PGRMC2, PHACTR1, PHACTR2, PHEX, PHF13, PHF2, PHF20, PIK3CD, PIK3IP1, PIK3R1, PIK3R5, PIP4K2A, PIP5K1B, PITPNM2, PKMYT1, PKNOX2, PLA2G15, PLA2G2F, PLAGL2, PLCD1, PLCE1, PLEK, PLEKHB1, PLEKHG3, PLEKHM3, PLEKHO2, PLIN1, PLXNA1, PLXNA2, PLXNA4, PMM2, PMP2, PMP22, PMPCB, PODN, POFUT1, POLD3, POLE4, POLR3H, PPARA, PPARGC1A, PPIG, PPM1A, PPP1R12B, PPP1R13L, PPP1R16B, PPP1R1A, PPP1R3D, PPP2R2A, PPP2R2D, PPP2R5C, PRDM15, PRDM16, PRDM2, PRF1, PRIM1, PRKAA1, PRKAA2, PRKAB2, PRKAG2, PRKAR2A, PRLR, PRND, PROP1, PRPF18, PRRG1, PSEN1, PSKH1, PSMB5, PSPH, PTBP1, PTGIR, PTGIS, PTK7, PTP4A1, PTPN11, PTPRR, PTPRS, PURA, PXDC1, PXMP2, PYCR1, PYGB, QKI, R3HCC1, RAB11FIP1, RAB11FIP4, RAB2A, RAB35, RAB37, RAB3C, RAB3IP, RAB40C, RAB43, RAB7A, RAB9B, RABIF, RABL5, RABL6, RAD17, RAD21, RAD51B, RAD54B, RALGAPA2, RALGAPB, RANBP17, RAP2A, RAP2B, RAPGEF3, RASAL2, RASD2, RAVER1, RBBP4, RBBP9, RBFOX3, RBM14, RBM20, RBM33, RBM4, RDX, RELT, REN, RFT1, RGAG1, RGS5, RHBDL1, RHOBTB2, RIC3, RILP, RIMBP2, RIMKLA, RLIM, RNF144A, RNF157, RNF213, RNF38, RNMT, ROR1, ROR2, RORA, RPA1, RPAP3, RPGR, RPL10, RPL27A, RPL28, RPL31, RPN1, RPP14, RPS14, RPS3, RRM2, RSPO4, RTKN, RTP1, RWDD2B, RXRA, RYR3, S100A11, S100A7A, SC5D, SCAI, SCN8A, SCO1, SCRT2, SDAD1, SDC1, SEC14L2, SELE, SEMA6A, SEMA6C, SEMG1, SERINC2, SERINC5, SERPINB5, SERPINB8, SERPINE1, SESN3, SETD1B, SFRP4, SFTPA1, SFTPA2, SGCD, SGPL1, SGPP2, SH3BGRL3, SH3BP2, SH3PXD2A, SH3PXD2B, SHC3, SHISA7, SHISA9, SHPRH, SHROOM4, SIK1, SIM1, SIM2, SIRPG, SIRT2, SIVA1, SKP1, SKP2, SLA, SLC12A2, SLC12A3, SLC13A1, SLC13A3, SLC13A5, SLC1A2, SLC1A3, SLC23A2, SLC25A23, SLC25A45, SLC35E1, SLC38A1, SLC38A10, SLC39A7, SLC3A2, SLC4A8, SLC5A3, SLC6A17, SLC6A7, SLC7A6, SLCO2A1, SLCO2B1, SLFN14, SMAD3, SMIM7, SMOC1, SMPD4, SNAI3, SNAP29, SNF8, SNTB2, SNX3, SNX5, SOCS7, SOD3, SOGA3, SORCS3, SORD, SORL1, SOS1, SOS2, SOWAHA, SOX12, SOX8, SP1, SP140, SP140L, SPATA13, SPATA5, SPECC1L, SPIB, SPOCK2, SPPL2A, SPPL2B, SPRYD3, SPTB, SREBF2, SRF, SRL, SRSF1, SRSF10, SRSF2, SSH1, SSH2, SSPN, ST5, ST7, ST8SIA2, ST8SIA3, STC2, STK10, STK17A, STK4, STON1, STOX2, STRA6, STS, STX1A, STXBP5L, SUFU, SUN2, SURF4, SUV39H1, SYNDIG1L, SYPL2, SYT12, SYT13, SZRD1, SZT2, TACR2, TADA2A, TAZ, TBC1D16, TBC1D22B, TBC1D24, TBC1D28, TBC1D30, TBCD, TCF3, TDRD6, TEC, TENM4, TESK1, TET2, TFAP2B, TFAP2C, TFAP4, TFB1M, TFCP2L1, TFDP2, TFEB, TGFA, TGFBR1, TGFBR2, THBS2, THEM4, THRB, TIA1, TIGD3, TIGIT, TIMM22, TIRAP, TK2, TLDC2, TLN2, TLR5, TMBIM6, TMC8, TMCO1, TMEM108, TMEM119, TMEM129, TMEM184B, TMEM192, TMEM194A, TMEM201, TMEM213, TMEM215, TMEM221, TMEM236, TMEM237, TMEM255A, TMEM261, TMEM92, TMTC1, TMUB2, TNFAIP1, TNFRSF1A, TNFRSF1B, TNFRSF21, TNFRSF25, TNFRSF8, TNFSF8, TNIK, TNNI1, TNNT3, TNPO3, TOB2, TOR1A, TOR1B, TP53I11, TP53INP1, TP53INP2, TP63, TPGS2, TPI1, TRABD2B, TRAF4, TRAF6, TRAPPC9, TRIAP1, TRIM25, TRIM39, TRIM45, TRIM5, TSPAN11, TSPYL1, TSPYL4, TSPYL5, TSSK2, TSTD2, TTBK2, TTC14, TTLL3, TUFT1, TULP3, TUSC2, TUSC3, TUSC5, TWIST1, TXNL4A, TYSND1, UBE2B, UBE2G1, UBE2H, UBE2J2, UBE2L3, UBE2O, UBE2R2, UBE2W, UBN2, UBOX5, UBTF, UMPS, UNC13A, UNC5D, UNC80, UPF1, UQCRB, USB1, USP13, USP22, USP36, USP46, USP47, UST, VAC14, VANGL1, VAPB, VASH1, VASP, VAV2, VGLL3, VHL, VPS26B, VPS52, VPS53, VPS72, VSTM2L, VSX2, WASF3, WBP1L, WDFY2, WDFY3, WDHD1, WDR48, WDR81, WEE1, WHSC1, WIPF2, WNK3, WNT11, WNT3, WSCD1, XDH, XK, XPC, XPO1, XPO5, YPEL1, YPEL2, YWHAZ, YY1, YY1AP1, ZBTB16, ZBTB20, ZBTB41, ZBTB46, ZBTB47, ZC3H12B, ZC3H12D, ZCCHC24, ZCCHC6, ZDHHC14, ZDHHC22, ZEB1, ZFP36, ZFX, ZHX3, ZKSCAN8, ZNF106, ZNF121, ZNF124, ZNF132, ZNF135, ZNF14, ZNF140, ZNF141, ZNF143, ZNF154, ZNF189, ZNF195, ZNF207, ZNF208, ZNF22, ZNF233, ZNF24, ZNF268, ZNF286A, ZNF33A, ZNF33B, ZNF440, ZNF445, ZNF451, ZNF490, ZNF496, ZNF500, ZNF540, ZNF554, ZNF555, ZNF559, ZNF584, ZNF589, ZNF592, ZNF641, ZNF652, ZNF660, ZNF697, ZNF70, ZNF704, ZNF720, ZNF728, ZNF74, ZNF747, ZNF761, ZNF772, ZNF780A, ZNF780B, ZNF814, ZNF844, ZSCAN2, ZSCAN20, ZSCAN26 |

List of predicted target genes of miR-6126 were obtained by using the miRWalk 2.0.
