# Supplementary material for: MicroRNA profiling in adults with high-functioning autism spectrum disorder
Source: Mol Brain. 2019 Oct 21;12:82. doi: 10.1186/s13041-019-0508-6 (PMC6802322; doi:10.1186/s13041-019-0508-6)
Supplement: Supplementary file 5 — Additional file 5: Table S5. GO and KEGG pathway analysis of target genes of miR-6126. [file 13041_2019_508_MOESM5_ESM.docx]

**Table S5. GO and KEGG pathway analysis of target genes of miR-6126.**

| **GO: Biological process** | ***p*-value** | **Genes** |
| --- | --- | --- |
| Neuron projection guidance (GO:0097485) | 2.79E-04 | ITGB1;ARX;NRXN3;AGAP2;LAMC1;EGFR;NRAS;EFNB3;OPHN1;GBX2;PLXNA2;ABL1;PLXNA1;WNT3;PLXNA4;CAP1;VASP;SEMA6C;SEMA6A;MYO10;DCC;EPHA8;ST8SIA2;PAX6;GFRA1;PTPN11;UNC5D;ANK3;SPTB;VAV2;ENAH;NFASC;CREB1;CXCL12;SCN8A;KCNQ2;DCX;KCNQ3;ISPD;SOS1;SOS2;FGFR1 |
| Axon guidance (GO:0007411) | 2.79E-04 | ITGB1;ARX;NRXN3;AGAP2;LAMC1;EGFR;NRAS;EFNB3;OPHN1;GBX2;PLXNA2;ABL1;PLXNA1;WNT3;PLXNA4;CAP1;VASP;SEMA6C;SEMA6A;MYO10;DCC;EPHA8;ST8SIA2;PAX6;GFRA1;PTPN11;UNC5D;ANK3;SPTB;VAV2;ENAH;NFASC;CREB1;CXCL12;SCN8A;KCNQ2;DCX;KCNQ3;ISPD;SOS1;SOS2;FGFR1 |
| Protein autophosphorylation (GO:0046777) | 4.21E-04 | CAMK2B;NTRK2;CSF1R;CAMK2D;FLT1;CDKL5;EPHA8;NEK6;INSR;CAMK2A;DAPK3;AATK;IRAK3;STK4;EGFR;ERN1;STK10;WNK3;AAK1;SIK1;TNIK;TSSK2;MAP3K11;FGFR1 |
| Protein stabilization (GO:0050821) | 4.77E-04 | USP13;CRTAP;SMAD3;PEX19;UBE2B;ATP1B2;A1CF;CREB1;LAMP2;ZNF207;AAK1;MDM4;TCF3;VHL;PPARGC1A;GPIHBP1 |
| Peptidyl-tyrosine phosphorylation (GO:0018108) | 5.33E-04 | NTRK2;CSF1R;FLT1;EPHA8;INSR;LIF;TESK1;AATK;PRLR;EGFR;WEE1;TEC;ABI2;PTK7;ABL1;ROR1;ROR2;MAP2K7;MAP2K5;MAP3K11;IL12RB2;FGFR1 |
| Peptidyl-tyrosine modification (GO:0018212) | 6.24E-04 | CSF1R;NTRK2;FLT1;EPHA8;INSR;LIF;TESK1;AATK;PRLR;EGFR;WEE1;TEC;ABI2;PTK7;ABL1;ROR1;ROR2;MAP2K7;MAP2K5;MAP3K11;IL12RB2;FGFR1 |
| Stress fiber assembly (GO:0043149) | 7.54E-04 | ITGB1;SRF;CUL3;PHACTR1;ARRB1;TNFAIP1 |
| Carnitine shuttle (GO:0006853) | 8.73E-04 | CPT1A;PRKAB2;PRKAA2;PRKAG2;ACACA |
| Amino acid transport (GO:0006865) | 9.52E-04 | NTRK2;SLC38A1;PRKAB2;CPT1A;PRKAA2;SLC6A17;SLC1A2;SLC1A3;SLC38A10;PRKAG2;SLC3A2;PSEN1;ACACA;SLC6A7;SLC7A6;XK;SERINC2;STX1A;SERINC5 |
| Negative regulation of locomotion (GO:0040013) | 9.87E-04 | PTPRR;SRF;SERPINE1;ADARB1;LDLRAD4;PBLD;SNX3;GPR173;WNT11;TRIM5;TP53INP1;TRIM25;WNT3;SCAI;MAP2K5;IGFBP5;DAB2IP;BRAF;VASH1;PODN;MCC;RAP2A;CXCL12;RAP2B;MMRN2;ADA |

| **GO: Cellular component** | ***p*-value** | **Genes** |
| --- | --- | --- |
| Axon initial segment (GO:0043194) | 3.16E-04 | CAMK2D;NFASC;SCN8A;KCNQ2;KCNQ3;ANK3 |
| Nucleoplasm (GO:0005654) | 6.38E-04 | THRB;NUMA1;PHF20;PRKAG2;PPP2R2A;RORA;ADARB1;NR2E1;NOC2L;CDC14B;PTBP1;ING5;RBM4;XPO1;PCF11;VPS72;HNF4A;XPO5;UBTF;DGCR8;KPNA4;NUDT16;SKP2;TXNL4A;TP63;SKP1;WDHD1;PRKAB2;NCBP2;MSL2;YWHAZ;CDC25A;SREBF2;RAD51B;PPM1A;KAT2A;CREB1;TFDP2;SRSF2;PGR;PPIG;PPARA;PPP1R12B;L3MBTL1;CAMK2B;CAMK2D;PRKAA2;RNMT;PRIM1;SP140;CAMK2A;SRSF1;NEDD4L;XPC;NR2C2;FOXO3;PKMYT1;HDAC9;POLD3;NXF1;ATXN3;ATXN1;RXRA;PSMB5;RBBP4;RAD21;E2F2;VHL;SRSF10;PPARGC1A;NR2C2AP;NKX2-2;CDT1;ZNF143;CBX5;SMAD3;RRM2;BRF1;SIVA1;FUS;CBX2;NFATC3;RPA1;FANCC;MAPK14;A1CF;MAPK11;WEE1;SP1;CENPI;KANSL3;RAD17;POLR3H;TCF3;CENPQ |
| Receptor complex (GO:0043235) | 1.35E-03 | GRIA1;ITGB1;CSF1R;FLT1;RNMT;GPR63;EGFR;CHRND;GRIN2A;CACNG8;LRRTM4;PLXNA2;PLXNA1;OLR1;PLXNA4;CACNG4;NTRK2;SMAD3;FCRL5;GRID1;INSR;IFNLR1;SHISA9;TGFBR1;GABRG1;TNFRSF1A;TGFBR2;TRAF6;ROR1;IL6ST;FGFR1;KCTD16 |
| Axon part (GO:0033267) | 4.01E-03 | GRIA1;NTRK2;CAMK2D;KCNA2;DAB2IP;SLC1A2;AATK;ANK3;TNFRSF1B;SIRT2;EEA1;NFASC;OPHN1;SCN8A;KCNQ2;TBC1D24;KCNQ3;AAK1 |
| AMP-activated protein kinase complex (GO:0031588) | 5.90E-03 | PRKAB2;PRKAA1;PRKAR2A;PRKAG2 |
| Node of Ranvier (GO:0033268) | 6.93E-03 | NFASC;SCN8A;KCNQ2;KCNQ3;ANK3 |
| Endosome membrane (GO:0010008) | 6.95E-03 | VAC14;SPPL2B;INSR;SPPL2A;IRAK4;LDLRAD4;CYB561A3;EGFR;HLA-E;SNX3;CLCN6;EHD2;CLCN5;RAP2A;ACAP2;RAP2B;TRAF6;LAMP2;RAB35;RAB11FIP4;SNX5 |
| Extrinsic component of membrane (GO:0019898) | 1.09E-02 | ARSA;MTMR3;TOR1A;EPB41;RDX;MSN;GFRA1;EEA1;EPB41L4A;EPB41L1;AAK1;ESYT3;WDFY3;CYTH1;SNX5 |
| Leading edge membrane (GO:0031256) | 1.23E-02 | GRIA1;ITGB1;APC2;CDKL5;PLEK;SLC1A2;SHISA9;TIRAP;SFRP4;ADAM17;SPATA13;RPS3;ARHGEF2 |
| Cytosol (GO:0005829) | 1.38E-02 | RAB3C;MTRR;RPL31;WIPF2;TESK1;PPP2R2A;BEST1;CNDP2;RPS14;ZFP36;OPHN1;GRAP2;PIP4K2A;PLCE1;TRIM25;KPNA4;SKP2;ATP6V1E1;TP63;PSPH;SKP1;EPM2A;PRKAB2;CTNNBIP1;LARP4B;IPCEF1;CDC25A;HGS;RPL28;CLOCK;PFKFB2;MTMR3;SHC3;NEDD4L;VPS26B;PIK3R1;FOXO3;STK4;PKMYT1;ACACA;NPAS2;TTBK2;PIK3R5;RHOBTB2;INPP5B;DYNC2LI1;SNX3;ATXN3;NDOR1;KIF3B;PCBP3;EPB41L1;PRKAR2A;RAD21;BLOC1S3;ABL1;THEM4;PIP5K1B;MAP2K7;PPARGC1A;EVI5;MAP2K5;VASP;CDT1;SMAD3;OSBPL5;BRPF3;NEK6;ZBTB16;PMM2;RAB3IP;NFATC3;FANCC;BRAF;SNF8;DAK;AGO1;RPL27A;NMT1;ADA;MOCS2;NUMA1;PLEK;PIK3CD;PRKAG2;ARRB1;HTR2A;EEA1;ACTR1A;CA1;PCM1;XPO1;CASP10;XPO5;PGM3;CASP2;PHACTR1;UPF1;PTGIR;GPX2;TPI1;DST;NCBP2;DCC;ARHGEF17;KSR2;GAB2;IRAK4;SPTB;YWHAZ;SERPINB8;GNL1;SIRT2;SREBF2;TIRAP;TGFBR2;VAV2;ACTA2;ENAH;PPM1A;RAP2A;RAP2B;TRAF6;AKT1S1;TNNT3;DCX;TNFRSF25;IRF5;ALDOC;PLIN1;ARHGEF2;IRF6;UMPS;SOS1;MOCS1;SOS2;BIRC3;ARHGEF6;AMER1;CAMK2B;CAMK2D;PRKAA1;PDXK;PRKAA2;AHCY;HPGD;RPL10;PELI3;HDC;CAMK2A;CBL;NSL1;CAMKK1;GNAI2;NXF1;PSMB5;TP53INP2;TP53INP1;RPS3;TNNI1;ZNF106;VHL;BID;XDH;CYTH1;PLA2G2F;NTRK2;SLC12A3;PEX19;RRM2;MYO10;NOS3;NDE1;PTPN11;KLHL3;CFLAR;MAPK14;MAPK13;MAPK11;TEC;ACTC1;PC;TRIM39;ABI2;CENPI;POLR3H;CENPQ;PLCD1 |

| **KEGG pathway** | ***p*-value** | **Genes** |
| --- | --- | --- |
| Neurotrophin signaling pathway_Homo sapiens_hsa04722 | 1.49E-06 | CAMK2B;CAMK2D;SHC3;CAMK2A;PIK3CD;PIK3R1;PSEN1;FOXO3;PIK3R5;NRAS;ABL1;MAP2K7;MAP2K5;NTRK2;FRS2;PTPN11;BRAF;IRAK3;IRAK4;MAPK14;MAPK13;MAPK11;TRAF6;SOS1;SOS2 |
| Proteoglycans in cancer_Homo sapiens_hsa05205 | 1.72E-06 | ITGB1;CAMK2B;CAMK2D;CAMK2A;TWIST1;PIK3CD;FZD10;PIK3R1;CBL;EGFR;PIK3R5;NRAS;WNT11;PLCE1;IL12B;WNT3;FZD5;MMP2;RDX;MSN;FRS2;BRAF;PTPN11;ANK3;MAPK14;MAPK13;VAV2;MAPK11;TFAP4;SDC1;PPP1R12B;SOS1;SOS2;FGFR1 |
| FoxO signaling pathway_Homo sapiens_hsa04068 | 2.04E-05 | PRKAB2;PRKAA1;PRKAA2;SMAD3;INSR;AGAP2;PRKAG2;PIK3CD;BRAF;PIK3R1;MAPK14;FOXO3;FBXO32;STK4;EGFR;TGFBR1;MAPK13;TGFBR2;PIK3R5;MAPK11;NRAS;SKP2;SOS1;SOS2 |
| Chronic myeloid leukemia_Homo sapiens_hsa05220 | 6.95E-05 | SHC3;SMAD3;PIK3CD;PTPN11;BRAF;GAB2;PIK3R1;CBL;TGFBR1;TGFBR2;PIK3R5;NRAS;ABL1;E2F2;SOS1;SOS2 |
| Oxytocin signaling pathway_Homo sapiens_hsa04921 | 9.43E-05 | CAMK2B;PRKAA1;CAMK2D;PRKAA2;CAMK2A;PRKAG2;PIK3CD;PIK3R1;EGFR;RYR3;CAMKK1;PIK3R5;GNAI2;NRAS;CACNG8;MAP2K5;KCNJ2;CACNG4;GUCY1A2;KCNJ5;PRKAB2;NOS3;CACNA2D1;NFATC3;PPP1R12B |
| Chagas disease_Homo sapiens_hsa05142 | 1.28E-04 | SMAD3;SERPINE1;PIK3CD;PPP2R2A;PIK3R1;CFLAR;IRAK4;MAPK14;TGFBR1;MAPK13;TGFBR2;TNFRSF1A;PIK3R5;GNAI2;MAPK11;GNAL;TRAF6;PPP2R2D;IL12B |
| AMPK signaling pathway_Homo sapiens_hsa04152 | 1.48E-04 | RAB2A;PFKFB2;PRKAB2;PRKAA1;CPT1A;PRKAA2;INSR;PRKAG2;PIK3CD;PPP2R2A;PIK3R1;PPP2R5C;FOXO3;ACACA;CAMKK1;PIK3R5;CREB1;HNF4A;AKT1S1;PPP2R2D;PPARGC1A |
| Non-small cell lung cancer_Homo sapiens_hsa05223 | 2.03E-04 | TGFA;PIK3CD;BRAF;PIK3R1;FOXO3;STK4;EGFR;PIK3R5;NRAS;RXRA;E2F2;SOS1;SOS2 |
| Glioma_Homo sapiens_hsa05214 | 2.29E-04 | CAMK2B;CAMK2D;SHC3;CAMK2A;TGFA;PIK3CD;BRAF;PIK3R1;EGFR;PIK3R5;NRAS;E2F2;SOS1;SOS2 |
| ErbB signaling pathway_Homo sapiens_hsa04012 | 4.05E-04 | CAMK2B;CAMK2D;SHC3;CAMK2A;TGFA;PIK3CD;BRAF;PIK3R1;CBL;EGFR;PIK3R5;NRAS;ABL1;SOS1;MAP2K7;SOS2 |

Both analyses were performed using the Enrichr database.
