# Supplementary material for: MicroRNA profiling in adults with high-functioning autism spectrum disorder
Source: Mol Brain. 2019 Oct 21;12:82. doi: 10.1186/s13041-019-0508-6 (PMC6802322; doi:10.1186/s13041-019-0508-6)
Supplement: Supplementary file 6 — Additional file 6: Figure S1. Blood cell composition. The proportions of six blood cell subtypes [B cells, CD8 T cells, CD4 T cells, natural killer (NK) cells, monocytes, and granulocytes] were estimated by Houseman’s algorithm using DNA methylation array data. A Student’s t-test was used for statistical analysis. [file 13041_2019_508_MOESM6_ESM.docx]

**Figure S1.** **Blood cell composition.**

The proportions of six blood cell subtypes [B cells, CD8 T cells, CD4 T cells, natural killer (NK) cells, monocytes, and granulocytes] were estimated by Houseman’s algorithm using DNA methylation array data. A Student's t-test was used for statistical analysis.

**
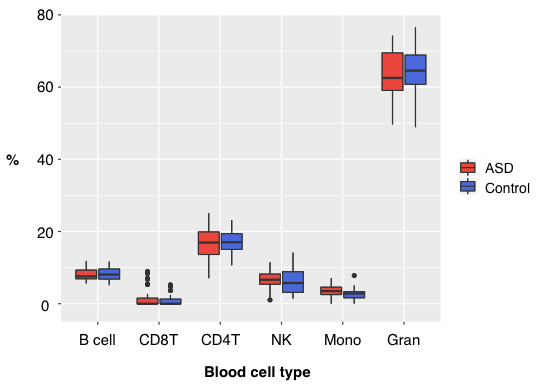
**
