# Supplementary material for: MicroRNA profiling in adults with high-functioning autism spectrum disorder
Source: Mol Brain. 2019 Oct 21;12:82. doi: 10.1186/s13041-019-0508-6 (PMC6802322; doi:10.1186/s13041-019-0508-6)
Supplement: Supplementary file 7 — Additional file 7. Materials and Methods. [file 13041_2019_508_MOESM7_ESM.docx]

**Methods**

**Participants**

A total of 30 patients with ASD were recruited from the Department of Psychiatry at Kyoto University Hospital along with 30 age- and gender- matched healthy individuals from among community volunteers. In addition to the Diagnostic and Statistical Manual of Mental Disorders, Fifth Edition (DSM-5) criteria, all participants were assessed using the Wechsler Adult Intelligence Scale [1], the Autism Diagnostic Observation Scale [2], and the Social Responsiveness Scale [3]. All participants were Japanese and took no medication at least three months prior to the collection of blood samples. This study was approved by the ethics committee of our institution, and written informed consent was obtained from all participants.

**Blood miRNA extraction**

MiRNAs were extracted from the peripheral blood of participants using a Paxgene Blood miRNA system (Qiagen, Tokyo, Japan). RNA quality and the presence of miRNA were confirmed using an Agilent 2100 Bioanalyzer (Agilent Technologies, Tokyo, Japan).

**MiRNA microarray**

Profiling of microRNA expression was performed using the SurePrint G3 Human miRNA 8x60K Microarray (G4870C) (Agilent Technologies) as previously described [4]. This microarray was designed to provide probes with both sequence and size discrimination, and to yield highly specific detection of closely related mature miRNAs [5]. Briefly, 100 ng of total RNA was labeled, hybridized, and scanned. The array platform contains 2549 human miRNAs based on miRBase 21.0. The data were processed and analyzed using the AgiMicroRna R-package [6]. After normalization and filtering out of poor-quality probes, 404 miRNAs were used in further analysis. A linear model was fitted to each miRNA, and differentially expressed miRNAs were considered as those with a *p*-value <0.01 and |fold change| > 1.5. Multiple testing correction based on the false discovery rate was also obtained by using the Benjamini-Hochberg procedure [7].

**MiRNA quantitative reverse transcription-polymerase chain reaction (qRT-PCR) analysis**

Total miRNAs from human bqrain, heart, kidney, liver, lung and spleen were purchased from BioChain (Newark, CA, USA). TaqMan MicroRNA Assays (Thermo Fisher Scientific, Yokohama, Japan) were used: has-miR-6126 (TaqMan Assay ID: 475618_mat, miRbase accession ID: MIMAT0024599) and U6 snRNA (TaqMan Assay ID: 0001973, miRbase accession ID: NR_004394). Briefly, 50 ng of total RNA was reverse transcribed, followed by pre-amplification with the TaqMan PreAmp Master Mix (Thermo Fisher Scientific). Real-time PCR reactions were run in duplicates on an ABI 7900HT Fast Real-Time PCR System (Thermo Fisher Scientific) and expression level was normalized to that of U6 snRNA.

**Machine learning analysis**

We employed a greedy algorithm, the Coarse Approximation Linear Function (CALF), to select potential miRNA biomarker candidates as previously described [8]. Briefly, this algorithm selected the one miRNA that best distinguished ASD from controls, based on the p-value, then added another miRNA that improved the overall p-value. The weights of selected miRNAs were either +1 or −1. This algorithm sought to add miRNAs until no further improvement in the *p*-value could be obtained. Then, to validate the statistical significance of selected miRNAs, this process was applied 2000 times to randomized data (randomly permuted ASD/control labels).

**Target Gene Prediction and Enrichment Analysis**

We searched the target genes of miR-6126 using miRWalk 2.0, which integrates multiple established target prediction tools [9]. The genes predicted by at least four algorithms (miRWalk, miRanda, RNAhybrid and TargetScan) were considered as reliable miRNA target genes. Gene ontology (GO) and Kyoto Encyclopedia of Genes and Genomes (KEGG) pathway analysis of target genes were performed using Enrichr [10]. To explore the relationship of target genes with ASD, we performed gene set enrichment analyses using two-sided Fisher's exact tests. The ASD Simons Foundation Autism Research Initiative (SFARI) genes were selected from the SFARI gene database (updated June, 2016) and filtered according to category S (syndromic) or evidence levels 1–4 [11]. Genes with protein-disrupting or missense rare de novo variants in ASD cases (de novo variants in ASD) were those compiled by Werling et al. [12].

**Estimation of White Blood Cell Composition**

To estimate blood cell composition, we used data from the Illumina Infinium HumanMethylation450 BeadChip (we note that some parts of these data have been submitted elsewhere for publication). Briefly, we collected blood samples for DNA and miRNA at the same time points. For DNA, whole blood samples were collected in EDTA tubes and genomic DNA was extracted using a QIAamp DNA Blood Midi Kit (Qiagen, Tokyo, Japan); and 500 ng of isolated DNA was bisulfite converted using an EZ DNA Methylation kit (Zymo Research, Irvine, CA). DNA methylation analyses were performed following the manufacturer’s protocol. Data quality control and analysis were performed using the ChAMP R-package (version 2.6.4) [13]. The blood cell composition was estimated using Houseman's algorithm in R [14]. Student's t-tests or Mann–Whitney U-tests were used to assess differences among the groups.

**References**

1. Ryan JJ, Carruthers CA, Miller LJ, Souheaver GT, Gontkovsky ST, Zehr MD. Exploratory factor analysis of the Wechsler Abbreviated Scale of Intelligence (WASI) in adult standardization and clinical samples. Appl Neuropsychol. 2003;10:252-6.

2. Lord C, Rutter M, Goode S, Heemsbergen J, Jordan H, Mawhood L, et al. Autism diagnostic observation schedule: a standardized observation of communicative and social behavior. J Autism Dev Disord. 1989;19:185-212.

3. Constantino JN, Davis SA, Todd RD, Schindler MK, Gross MM, Brophy SL, et al. Validation of a brief quantitative measure of autistic traits: comparison of the social responsiveness scale with the autism diagnostic interview-revised. J Autism Dev Disord. 2003;33:427-33.

4. Kimura R, Swarup V, Tomiwa K, Gandal MJ, Parikshak NN, Funabiki Y, et al. Integrative network analysis reveals biological pathways associated with Williams syndrome. J Child Psychol Psychiatry. 2019;60:585-98.

5. Wang H, Ach RA, Curry B. Direct and sensitive miRNA profiling from low-input total RNA. RNA. 2007;13:151-9.

6. López-Romero P. Pre-processing and differential expression analysis of Agilent microRNA arrays using the AgiMicroRna Bioconductor library. BMC Genomics. 2011;12:64.

7. Benjamini Y, Hochberg Y. Controlling the false discovery rate: a practical and powerful approach to multiple testing. J Royal Stat Soc B (Methodological). 1995;57:289-300.

8. Jeffries CD, Perkins DO, Chandler SD, Stark T, Yeo E, Addington J, et al. Insights into psychosis risk from leukocyte microRNA expression. Transl Psychiatry. 2016;6:e981.

9. Dweep H, Gretz N. miRWalk2.0: a comprehensive atlas of microRNA-target interactions. Nat Methods. 2015;12:697.

10. Chen EY, Tan CM, Kou Y, Duan Q, Wang Z, Meirelles GV, et al. Enrichr: interactive and collaborative HTML5 gene list enrichment analysis tool. BMC Bioinformatics. 2013;14:128.

11. Banerjee-Basu S, Packer A. SFARI Gene: an evolving database for the autism research community. Dis Model Mech. 2010;3:133-5.

12. Werling DM, Parikshak NN, Geschwind DH. Gene expression in human brain implicates sexually dimorphic pathways in autism spectrum disorders. Nature Commun. 2016;7:10717.

13. Tian Y, Morris TJ, Webster AP, Yang Z, Beck S, Feber A, et al. ChAMP: updated methylation analysis pipeline for Illumina BeadChips. Bioinformatics. 2017;33:3982-4.

14. Houseman EA, Accomando WP, Koestler DC, Christensen BC, Marsit CJ, Nelson HH, et al. DNA methylation arrays as surrogate measures of cell mixture distribution. BMC Bioinformatics. 2012;13:86.
